# Heineke-Mikulicz pyloroplasty for isolated pyloric stricture caused by corrosive ingestion in children

**DOI:** 10.12669/pjms.36.ICON-Suppl.1714

**Published:** 2020-01

**Authors:** Muhammad Aqil Soomro, Maryam Aftab, Maria Hasan, Hana Arbab

**Affiliations:** 1 Dr. Muhammad Aqil Soomro, FCPS. Division of Pediatric Surgey, The Indus Hospital, Karachi, Pakistan; 2 Dr. Maryam Aftab, FCPS. Division of Pediatric Surgey, The Indus Hospital, Karachi, Pakistan; 3 Dr. Maria Hasan, MBBS. Division of Pediatric Surgey, The Indus Hospital, Karachi, Pakistan; 4 Dr. Hana Arbab, FCPS. Division of Pediatric Surgey, The Indus Hospital, Karachi, Pakistan

**Keywords:** Corrosive, Children, Pyloric stricture, Gastric outlet obstruction, Heineke-Mikulicz pyloroplasty

## Abstract

Corrosive ingestion in children is a common problem in low income countries. These agents cause injuries and later strictures of esophagus and stomach. Gastric outlet obstruction is known complication of acids and surgery is the mainstay of treatment. There are multitude of surgical options for these strictures depending on the involved segment of the stomach and experience of the surgeon. Here we present three cases of children who accidentally ingested acid stored in soda bottles and subsequently developed isolated pyloric strictures. These cases presented between August 2018 and April 2019 to our facility, a tertiary care hospital in Karachi, Pakistan. All three patients had an initial latent period of one to two weeks following corrosive ingestion, after which symptoms of gastric outlet obstruction appeared. Intraoperatively, all three had normal esophagus and antrum but scarred and strictured pylorus. Heineke-Mikulicz pyloroplasty was done in these cases without complications and the outcomes were satisfactory.

## INTRODUCTION

Accidental corrosive ingestion in the pediatric age group is quite common in low middle income countries (LMICs), because these agents are readily available in unsafe packing and easily accessible to children in households. As opposed to alkali ingestion which results primarily in oropharyngeal and esophageal damage, acids tend to spare the esophagus and produce coagulation necrosis of the stomach.^1^ Stomach injury usually occurs in the antrum and pylorus as the acid follows the lesser curvature of the stomach to pool in the antrum due induced reflex pylorospasm.^2^ The most sinister sequelae of these injuries is stricture formation causing gastric outlet obstruction.

The exact prevalence and incidence of these injuries in Pakistan is unknown and there is little local literature. Management of these strictures varies widely from endoscopic dilatation, gastrojejunostomy, Billroth-1 partial gastrectomy, Heineke-Mikulicz pyloroplasty, Finney pyloroplasty and antroplasty, depending upon the severity and location of injuried stomach.^3^-^5^ We report three cases of isolated pyloric stricture caused by corrosive ingestion managed at The Indus Hospital, Karachi (TIH) between August 2018 and April 2019.

## CASE-1

A one year and eleven months old boy weighing 7.2 kilograms (kgs) was referred to Emergency with complaints of vomiting after corrosive ingestion one month previously. The child drunk from a soda bottle in which toilet cleaner had been stored. He was initially taken to a local hospital in his native town from where he was discharged after 24 hours of observation and good oral intake. He started vomiting 10 days after ingestion. His upper GI contrast study showed near total obstruction for which surgery was advised and the child was referred to TIH. Here we performed fiberoptic endoscopy after initial assessment and workup. The esophagus and stomach appeared normal, but the pylorus was scarred and completely obliterated such that even a guidewire could not be passed. Subsequently, the family was counseled and surgery planned. Intraoperatively, an isolated pyloric stricture was identified whilst the duodenum and antrum were normal. Heineke-Mikulicz pyloroplasty was performed, leaving a trans-anastomotic jejunal tube through which feeding was started 24 hours later. The child was discharged three days later when he tolerated full feed. He was followed up and nine months post-surgery, he was tolerating oral diet well and gaining weight.

## CASE-2

**Fig.1:**
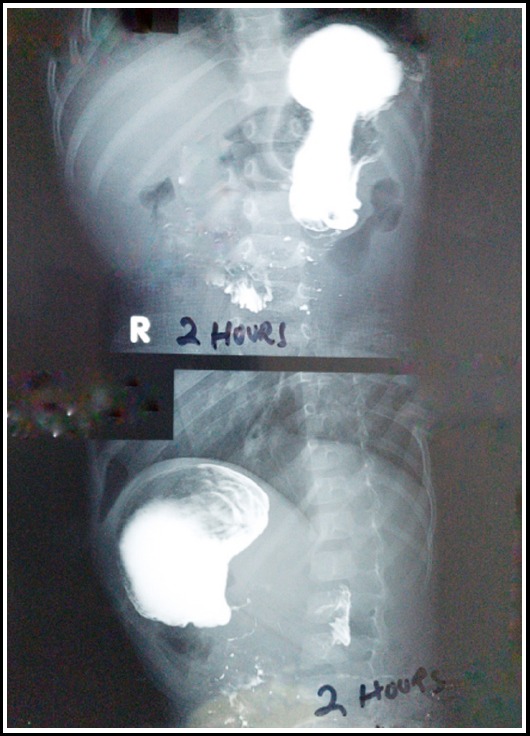
Contrast study showing complete obstruction at pylorus just trickle of contrast even after two hours.

A boy aged three years and eleven months weighing 9.5 kg was brought to ER with accidental ingestion of battery fluid (diluted sulfuric acid). It had mistakenly been consumed as it was stored in a soda bottle (Fig.1). Minor oral burns were noted and the child was crying excessively, for which he was brought to our Emergency immediately. Since no significant oropharyngeal injuries were seen on initial evaluation, oral feeds were started gradually and were tolerated. After 24 hours he was discharged, with advice to follow up immediately if dysphagia or vomiting was observed. He returned to TIH 15 days later with vomiting noted after feeding. An endoscopy done on the 22^nd^ day post ingestion revealed a normal esophagus and stomach, with a tight stricture at the pylorus. After counseling the family, surgery was planned. Per-operatively, an isolated, complete pyloric stricture was seen, with a normal antrum and duodenum. Heineke-Mikulicz pyloroplasty was performed, retaining a trans anastomotic nasojejunal tube in place, through which nasojejunal feeding was started 24 hours post-surgery. The child developed recurrent abdominal distension post feed with improvement over a few hours. An upper GI contrast study was done on the sixth postoperative day and found to have delayed gastric emptying. Conservative management was employed at this stage and he started feed gradually, reaching full feed on the 10^th^ postoperative day. Discharge was arranged with follow-up and 9 months post surgery, he was thriving and gaining weight.

## CASE-3

A three years and seven months-old girl weighing 5kg was referred to Emergency from a rural area, with a history of accidental battery fluid ingestion two months back. Family had stored battery fluid in a soda bottle. The child developed vomiting 10 days post ingestion for which she was taken to different hospitals without receiving any definitive treatment. During this time, she sustained significant weight loss. An upper GI contrast study was done in a peripheral hospital which demonstrated complete pyloric obstruction. She was then referred to TIH for further management. Since the child was emaciated, total parenteral nutrition was initially started. Subsequent fiberoptic endoscopy revealed a normal esophagus and stomach, but a scarred and strictured pylorus. After counseling, surgery was done which confirmed the presence of an isolated pyloric stricture with normal duodenum and antrum. Heineke-Mikulicz pyloroplasty was performed with a trans anastomotic nasojejunal tube left in place, through which feeding was started 24 hours later. She started oral feeds on fifth day and reached full feeds on the seventh postoperative day before eventual discharge. At her most recent outpatient followup, two months post procedure, she was gaining weight steadily on a full oral diet.

## DISCUSSION

Corrosive ingestion causing injury to the stomach was first reported in 1882 but it was not until 1962 that Karon described the pathophysiological events leading to pyloric stenosis in adult patients.^6^ Corrosive ingestion was followed by a latent period, typically one to two weeks, where patients may be asymptomatic. Antropyloric stenosis with gastric outlet obstruction symptoms develop subsequently. All three of our patients had a similar history of corrosive ingestion followed by an initial latent period and then the appearance of increasingly severe obstructive symptoms.

In Karachi, Siddiqui et al. found that 41% of cases of accidental corrosive ingestion, the substances were stored in beverage bottles.^7^ This was the case in all three children in our series. Public awareness on proper storage of corrosive agents is important. There should be education about clear labelling of dangerous substances and reduced accessibility to children. This can reduce the incidence of these accidental injuries and hence related morbidity and mortality.

Recommendations for optimal timing of surgical intervention in such patients vary. Early repair is proposed by Tekant et al.^8^ to prevent weight loss. Meanwhile, Chaudry et al. recommended late repair^9^ based on their understanding that the fibrotic process develops over a period of time, and maximum fibrosis should develop before intervening. We opted for early repair because all of our patients had total obstruction as evidenced by contrast study and no concomitant esophageal or gastric injuries visualized on fiberoptic endoscopy. Prolonged parenteral nutrition has inherent challenges and is not feasible in our patient population. Early surgical intervention enabled earlier feeding and prevented further weight loss and prolonged hospital stays.

There is a plethora of surgical procedures to choose from for corrosive injuries. In a recent report Seleim et al. used laparoscopic diamond shaped antroduodenostomy for pyloric strictures.^10^ In a local series for management of gastric outlet obstruction due to corrosive ingestion, Imran et al used Billroth 1 as their procedure of choice.^5^ This was based upon the findings of injuries and scarring of the antrum. In an Egyptian series by El-Asmar et al, a variety of procedures were performed in 26 patients, including gastrectomy, partial gastrectomy, Billroth 1, antrectomy, antroplasty, Finney pyloroplasty, Heineke-Mikulicz pyloroplasty and gastrojejunostomy.^3^ This reflects the need to choose the procedure on a case-by-case basis, depending on the involved segments of the stomach and experience of the surgeon. We opted for pyloroplasty in our cases because of the isolated involvement of pylorus. Successful outcomes were seen by the immediate return to oral diet and in medium-term, with good weight gain and continued tolerance of full oral diet.

## CONCLUSION

Accidental corrosive ingestion in children is not uncommon in pediatric age group in LMICs. These sinister liquids are commonly bought loose and kept in innocuous looking beverage bottles in homes to which children have easy access. Corrosives cause injury to the esophagus and/or stomach which may result in stricture formation. There are different surgical options available for management of these strictures. Heineke-Mikulicz pyloroplasty is an option in isolated pyloric stricture patients, which we have used with good outcomes.
